# Mpox Diagnosis, Behavioral Risk Modification, and Vaccination Uptake among Gay, Bisexual, and Other Men Who Have Sex with Men, United Kingdom, 2022

**DOI:** 10.3201/eid3005.230676

**Published:** 2024-05

**Authors:** Dana Ogaz, Qudsia Enayat, Jack R.G. Brown, Dawn Phillips, Ruth Wilkie, Danielle Jayes, David Reid, Gwenda Hughes, Catherine H. Mercer, John Saunders, Hamish Mohammed, UK Health Security Agency Sexual Health Liaison Group

**Affiliations:** UK Health Security Agency, London, UK (D. Ogaz, Q. Enayat, D. Phillips, R. Wilkie, D. Jayes, G. Hughes, J. Saunders, H. Mohammed);; The National Institute for Health and Care Research Health Protection Research Unit in Blood Borne and Sexually Transmitted Infections at University College London in partnership with the UK Health Security Agency, London, UK (D. Ogaz, J.R.G. Brown, D. Reid, G. Hughes, C.H. Mercer, J. Saunders, H. Mohammed);; University College London, London, UK (D. Reid, C.H. Mercer, J. Saunders);; London School of Hygiene & Tropical Medicine, London, UK (G. Hughes)

**Keywords:** Mpox, monkeypox virus, viruses, sexually transmitted infections, men who have sex with men, gay, homosexual, bisexual, vaccination, risk, behavior, risk modification, vaccine uptake, health equity, United Kingdom

## Abstract

During the 2022 multicountry mpox outbreak, the United Kingdom identified cases beginning in May. UK cases increased in June, peaked in July, then rapidly declined after September 2022. Public health responses included community-supported messaging and targeted mpox vaccination among eligible gay, bisexual, and other men who have sex with men (GBMSM). Using data from an online survey of GBMSM during November–December 2022, we examined self-reported mpox diagnoses, behavioral risk modification, and mpox vaccination offer and uptake. Among 1,333 participants, only 35 (2.6%) ever tested mpox-positive, but 707 (53%) reported behavior modification to avoid mpox. Among vaccine-eligible GBMSM, uptake was 69% (95% CI 65%–72%; 601/875) and was 92% (95% CI 89%–94%; 601/655) among those offered vaccine. GBMSM self-identifying as bisexual, reporting lower educational qualifications, or identifying as unemployed were less likely to be vaccinated. Equitable offer and provision of mpox vaccine are needed to minimize the risk for future outbreaks and mpox-related health inequalities.

On July 23, 2022, the World Health Organization (WHO) declared a Public Health Emergency of International Concern for the global mpox (formerly known as monkeypox) outbreak (*1*). By late March 2023, more than 85,000 confirmed mpox cases had been reported across 110 countries (*2*). Monkeypox virus, the cause of mpox, can be transmitted through close, personal contact with an infected person, including during sex (*1*). The mpox outbreak that began in 2022 was international, predominantly transmitted through sexual networks, and disproportionately affected gay, bisexual, and other men who have sex with men (GBMSM) (*1*,*3*–*6*).

Mpox was detected in the United Kingdom in May 2022, but community transmission was estimated to have started the previous month (*3*,*5*). UK case numbers peaked in July 2022 and reached >3,500 cases across the country by the end of 2022. The UK Health Security Agency (UKHSA) enacted measures to curb rising incidence, including raising public health awareness, advising a 21-day self-isolation period for persons with an mpox diagnosis, undertaking comprehensive contact-tracing of recent close sexual contacts, and recommending a targeted vaccination campaign using the modified vaccinia Ankara vaccine (*7*). UKHSA closely worked with community-based organizations to inform public health messaging to raise awareness and reduce the risk for mpox (*8*–*10*). An expert consensus panel that included UKHSA and national and community sexual health organizations estimated that 111,000 persons would be eligible for mpox vaccination in the United Kingdom, including 103,000 GBMSM and 8,000 healthcare and outreach workers (*11*). The vaccination campaign began in June 2022 and provided 70,837 first doses and 31,827 second doses by the end of May 2023 (*12*). UK case numbers rapidly subsided by the end of September 2022, and few cases were reported in 2023 (*13*).

Vaccine delivery was initially targeted to sexual health services (SHS) in London because 69% of cases through September 2022 were among residents of the city (*11*). National guidance recommended GBMSM at highest risk for mpox could be identified among persons seeking care at SHS by using similar markers of risk used to identify persons eligible for HIV preexposure prophylaxis (PrEP), irrespective of HIV status (*7*,*14*). Vaccine eligibility criteria included a history of multiple recent sexual partners, group sex, or attending sex-on-premises venues, or a diagnosis of a bacterial sexually transmitted infection (STI). Initially, first doses were offered to eligible SHS clients as a subcutaneous injection (*7*,*15*). Later, fractional dosing via intradermal administration also was pilot tested and rolled out to maximize the coverage of the available vaccine supply (*16*). Observational, real-world studies reported 78%–86% vaccine effectiveness in preventing symptomatic mpox (*17*,*18*).

In response to the UK mpox outbreak, UKHSA rapidly deployed the Reducing inequalities in Sexual Health (RiiSH)-Mpox survey during November 24–December 19, 2022. RiiSH-Mpox was designed to assess the effects of the mpox outbreak on the health, well-being, sexual behavior, and SHS use among a community sample of GBMSM in the United Kingdom. We used RiiSH-Mpox survey data to examine self-reported mpox diagnosis history, behavioral risk modification, and uptake of mpox vaccination among GBMSM in the United Kingdom.

## Methods

### Data Collection and Study Design

The RiiSH-Mpox survey was adapted from an established methodology used to deliver a series of cross-sectional surveys conducted during 2017 and later used during periods before and after COVID-19–related social restrictions in the United Kingdom (*19*,*20*). The RiiSH-Mpox survey included all previous questions on SHS use and sexual risk behavior but incorporated a novel module that was developed with community stakeholders and asked about mpox diagnosis, vaccination uptake, and behavioral risk modification in response to the outbreak. The UKHSA Research and Ethics Governance Group provided ethics approval for this study (reference no. R&D 524), and all methods were performed in accordance with guidelines and regulations set by that group. Online consent was obtained from all participants, and no incentive was offered to participate.

### Setting and Sampling

As in previous rounds of RiiSH surveys, RiiSH-Mpox recruited participants via advertisements on social networking sites, including Facebook (https://www.facebook.com), Instagram (https://www.instagram.com), and Twitter (https://www.twitter.com), as well as on Grindr (https://www.grindr.com), a geospatial networking (dating) application. The survey was conducted during November 24–December 19, 2022. 

Persons included in the analyses were >16 years of age, UK residents, and self-identifying as men (cisgender or transgender), transgender women, or gender-diverse persons assigned male at birth. Included persons also reported having had sex with a cisgender or transgender man or with a gender-diverse person assigned male at birth since November 2021. 

### Data Analysis

#### Mpox Testing and Diagnosis History

We calculated the percentage of persons reporting an mpox diagnosis history (i.e., positive mpox test) anytime through survey completion and used the Clopper-Pearson interval to calculate 95% CIs. As a sensitivity analysis and to quantify potentially undiagnosed infections, we calculated the percentage of persons reporting a diagnosis history, those with self-perceived mpox in absence of a positive mpox test, and those with self-reported testing history.

We used the Pearson χ^2^ test to assess differences in sociodemographic, clinical, and behavioral characteristics. Because so few participants had a diagnosis history, we did not conduct regression analyses.

#### Mpox Vaccination Uptake

We defined vaccination uptake as receipt of >1 mpox vaccine doses. We assessed report of vaccine offer (i.e., “Have you been offered a vaccine for monkeypox?”) among participants who reported no vaccine uptake. We calculated the percentage and 95% CI of uptake among participants offered an mpox vaccine and in all participants. We also assessed vaccine willingness for participants reporting they would likely or definitely take an mpox vaccine if offered and among participants who were not offered the vaccine.

#### Vaccination Uptake among Vaccine Eligible Participants

We examined uptake among participants assumed to be eligible for mpox vaccination on the basis of equivalent or proxy criteria outlined in national vaccination guidance (*14*). Using survey responses, we defined vaccine eligibility as the report of any of the following since August 2022: >10 physical male sex partners; meeting any physical male sex partner at a sex on premises venue, sex party, or cruising grounds (hereafter, public sex environment [PSE]); a positive STI test; or, in the past year, report of PrEP use (as a proxy for persons at higher risk for acquiring mpox) or use of recreational drugs associated with chemsex (i.e., crystal methamphetamine, mephedrone, or gamma-hydroxybutyrate/gamma-butyrolactone). As a sensitivity analysis to consider a less conservative measure of having multiple partnerships, we used a lower threshold of >5 physical male sex partners since August 2022, instead of >10, to define eligibility.

### Factors Associated with Mpox Vaccination

We assessed factors associated with mpox vaccination by using the Pearson χ^2^ test and binary logistic regression. We included sociodemographic variables that had a significant bivariate association with vaccination in multivariable regression models and sequentially assessed associations of clinical and behavioral characteristics with mpox vaccination. We selected age group and ethnicity a priori for inclusion in multivariable modeling. We also conducted a sensitivity analysis to examine sociodemographic factors associated with mpox vaccination among vaccine-eligible participants to assess potential uptake inequalities among that group.

We used the following sociodemographic characteristics in our analyses: age group, ethnicity, gender, sexual orientation, country of birth, nation of residence in the United Kingdom (England, Scotland, Wales, or Northern Ireland), education level, employment, household composition (living alone or not), relationship status (single or in a relationship), and report of a comfortable financial situation from the top 2 quartiles (e.g., a response of “I am comfortable” or “I am very comfortable” from the question “How would you best describe your current financial situation?”). We also used clinical characteristics in our multivariable analyses, including HIV status and uptake of >1 vaccine doses for hepatitis A virus (HAV), hepatitis B virus (HBV), or human papillomavirus (HPV). We defined behavioral risk modification as the report of any of the following beginning in May 2022: fewer sexual partners; reduced visits to sex on premises venues or PSE; or avoiding any sex, condomless anal sex, skin-to-skin contact, or visiting clubs or crowds. Last, we used the following sexual risk behaviors in our analyses: number and meeting place of male physical sex partners since August 2022, a positive STI test since August 2022, and report of PrEP or recreational drug use associated with chemsex during the previous year. Lookback intervals for sexual risk behaviors varied because the survey aimed to align timeframes used in prior RiiSH surveys or with timeframes from the start of the mpox outbreak in May 2022.

Because some subgroups had small participant numbers, we dichotomized some groups for analyses. Those groups included ethnicity, which we dichotomized to White and all other ethnic groups (non-White); gender, which we dichotomized to cisgender male and all other gender identity groups; and sexual orientation, which we dichotomized to gay or homosexual and bisexual, which included participants identifying as bisexual, straight, or “another way.” 

We collected survey data via the Snap Surveys platform (https://www.snapsurveys.com). We managed data and conducted analyses by using Stata version 15.0 (StataCorp LLC, https://www.stata.com). We considered p<0.05 statistically significant.

## Results

Among 1,435 GBMSM that engaged with the RiiSH-Mpox survey, 93% (1,333) met eligibility criteria (Figure). Missing data were limited (<3% item nonresponse) because most survey questions were compulsory. Median age among participants was 45 (range 16–78, interquartile range [IQR] 35–55) years (Table 1; Appendix Table 1). Most participants self-identified as cisgender male (99%), gay or homosexual (89%), of White ethnicity (92%), residents of England (86%), and employed (81%). Nearly half (48%) of participants reported a comfortable financial situation, 63% had degree-level qualifications, and 15% were living with HIV (Appendix Tables 1, 2). Most (58%) participants were recruited from Facebook, 24% were recruited from Grindr and 15% from Twitter. Among all participants, 53% (707/1,333) reported behavioral modification to avoid getting mpox, most (72% [510/707]) of whom reported reducing the number of physical male sex partners as a prevention measure (Table 2).

**Figure:**
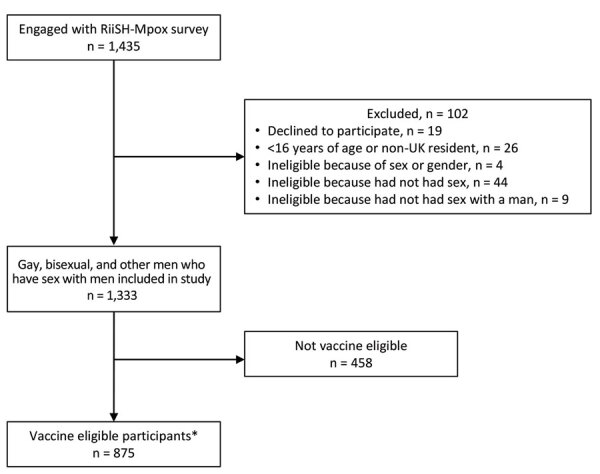
Flowchart of selection criteria in a survey of mpox diagnosis, behavioral risk modification, and vaccination uptake among gay, bisexual, and other men who have sex with men, United Kingdom, 2022. *Participants were eligible for mpox vaccination if they self-reported any of the following: meeting recent male physical sex partners at sex on premises venues, sex parties, or public sex environment (i.e., cruising grounds) since August 2022; >10 recent male physical sex partners since August 2022; recreational drug use associated with chemsex (e.g., crystal methamphetamine, mephedrone, or gamma-hydroxybutyrate/gamma-butyrolactone) in the past year; recent positive sexually-transmitted infection test since August 2022; or current HIV preexposure prophylaxis use since December 2021. RiiSH-Mpox, Reducing inequalities in Sexual Health Mpox survey.

**Table 1:** Sociodemographic and clinical characteristics of participants in a survey of mpox diagnosis, behavioral risk modification, and vaccination uptake among gay, bisexual, and other men who have sex with men, United Kingdom, 2022*

Characteristics	RiiSH-Mpox participants, no. (%)	Mpox diagnosis history, no. (%)	No mpox diagnosis history, no. (%)	p value
Total	1,333 (100)	35 (100)	1,298 (100)	
Recruitment site				
Facebook	769 (58)	28 (80)	741 (57)	
Grindr	316 (24)	5 (14)	311 (24)	
Twitter	198 (15)	2 (6)	196 (15)	
Instagram	18 (1)	0	18 (1)	
Other	32 (2)	0	32 (2)	0.099
Sociodemographic characteristics				
Median age, y (range; IQR)	45 (16–78; 35–55)	42 (25–58; 38–47)	45 (16–78; 35–55)	
Binary age group, y				
<35	319 (24)	3 (9)	316 (24)	
>35	1,014 (76)	32 (91)	982 (76)	0.031
Gender identity, sex at birth				
Cisgender male	1,314 (99)	30 (86)	1,193 (92)	
Other gender identity group	19 (1)	0	19 (1)	0.376
Sexual orientation				
Gay or homosexual	1,187 (89)	32 (91)	1,155 (89)	
Bisexual†	146 (11)	3 (9)	143 (11)	0.648
Ethnicity				
White	1,223 (92)	45 (87)	1,178 (92)	
Non-White	110 (8)	5 (14)	105 (8)	0.189
Country of birth				
United Kingdom	1,113 (83)	22 (63)	1,091 (84)	
Outside the United Kingdom	220 (17)	13 (37)	207 (16)	0.001
Nation of residence				
England	1,148 (86)	34 (97)	1,114 (86)	
Scotland, Wales, or Northern Ireland	185 (14)	1 (3)	184 (14)	0.056
Educational level				
Below degree level	495 (37)	7 (20)	488 (38)	
Degree level or higher	838 (63)	28 (80)	810 (62)	0.033
Clinical and behavioral characteristics				
HIV status				
Negative or unknown	1,131 (85)	22 (63)	1,109 (85)	
Living with HIV	202 (15)	13 (37)	189 (15)	0.001
STI tested in past year				
N	405 (30)	1 (3)	404 (31)	
Y	928 (70)	34 (97)	894 (69)	<0.001
Recreational drug use associated with chemsex in past year‡				
N	1,222 (92)	27 (77)	1,195 (92)	
Y	111 (8)	8 (23)	103 (8)	0.002
Number of recent male physical sexual partners since August 2022				
0	100 (8)	1 (3)	99 (8)	
1	180 (14)	2 (6)	178 (14)	
2–4	389 (29)	9 (26)	380 (29)	
5–9	280 (21)	6 (17)	274 (21)	
>10	384 (29)	17 (49)	367 (28)	0.094
Met recent male physical sex partners in sex on premises venue, sex party, or PSE since August 2022				
N	888 (67)	17 (49)	871 (67)	
Y	445 (33)	18 (51)	427 (33)	0.022
Behavior modification to avoid getting mpox since May 2022§				
N	626 (47)	18 (51)	608 (47)	
Y	707 (53)	17 (49)	690 (53)	0.592

*Participants with self-reported mpox test positivity. Those without mpox test positivity (i.e., no diagnosis history) include those without a known positive test (n = 67) and those without a test history (n = 1,231). Percentages may not sum to 100 due to rounding. PSE, public sex environment (i.e., cruising grounds); RiiSH-Mpox, Reducing inequalities in Sexual Health Mpox survey; STI, sexually transmitted infection. †Includes those identifying as bisexual, straight, or another orientation. ‡Includes crystal methamphetamine, mephedrone, or gamma-hydroxybutyrate/gamma-butyrolactone. §Behavior modification includes self-report of any of the following from May 2022: fewer sexual partners, reduced visits to sex on premises venues or PSE (i.e., cruising grounds), and avoiding: all sex, condomless anal sex, skin-to-skin contact, or visiting clubs or crowds.

**Table 2:** Behavior modification measures and other precautions reported among participants in a survey of mpox diagnosis, behavioral risk modification, and vaccination uptake among gay, bisexual, and other men who have sex with men, United Kingdom, 2022*

Behavior modification measure†	No. participants	% All participants, n = 1,333	% Reporting any modification measures, n = 707
Self-report of >1 behavior modification measure since May 2022 to avoid getting mpox	707	53	100
I've chosen fewer sexual partners	510	38	72
I've reduced visits to or avoided sex-on-premises venues such as saunas and backrooms	351	26	50
I've avoided sex	206	15	29
I've reduced visits to or avoided cruising grounds	185	14	26
I've avoided nightclubs or other crowded spaces	138	10	20
I've avoided condomless sex	106	8	15
I've avoided skin-to-skin contact	69	5	10
None of the above behavior modification measures reported	626	47	NA
Other precautions reported since May 2022§			
I've asked potential sex partners if they've had a monkeypox vaccination	187	14	NA
I've washed my hands more regularly	129	10	NA
I've asked potential partners if they've had mpox symptoms	124	9	NA
I've asked sexual partners for contact details for contact tracing	22	2	NA

*Behavior modifications were derived from the question, “Since the start of the UK monkeypox outbreak in May 2022, have you done any of the following in order to avoid getting monkeypox?” NA, not applicable. †Participants could select >1 behavioral modification measure listed; responses are not mutually exclusive. §Shown for information not included as behavioral modification measures in analysis; responses are not mutually exclusive.

### Mpox Diagnosis History

Among all 1,333 participants, 35 (2.6% [95% CI 1.8%–3.6%]) reported an mpox diagnosis history (i.e., mpox test positivity) (Table 1; Appendix Table 1, Figure 1). An additional 17 participants reported self-perceived mpox, including 3 who were never tested and 14 who were tested before survey completion, for a total of 52 participants (3.9% [95% CI 2.9%–5.1%]) reporting either mpox test positivity or self-perceived mpox (Appendix Table 3, Figure 1).

Compared with 1,298 participants without an mpox diagnosis history (i.e., no mpox test positivity), the 35 participants who had an mpox-positive test result were more likely to be >35 years of age (91% vs. 76%; p = 0.031), born outside the United Kingdom (37% vs. 16%; p = 0.001), in a comfortable financial situation (66% vs. 48%; p = 0.034), and living with HIV (37% vs. 15%; p<0.001) (Table 1; Appendix Table 1). Participants with an mpox diagnosis via testing also reported higher levels of SHS clinic engagement in the past year, recent STI test positivity, and sexual risk behaviors such as meeting partners at sex on premises venue, sex party, or PSE (Table 1; Appendix Table 1). Persons with and without an mpox diagnosis history reported similar proportions of outbreak-related behavior modification since May 2022 (49% vs. 53%; p = 0.592); those results were similar to findings on the sensitivity analysis (Appendix Table 3).

### Mpox Vaccination Uptake among All Participants

More than half (58%, 771/1,333) of participants were offered an mpox vaccination, and 692 received vaccination. Vaccination uptake was 52% (95% CI 49%–55%) for all participants and 90% (95% CI 87%–92%) for those who were offered a vaccine (Table 3; Appendix Table 2). Of participants reporting receiving vaccination, only 41% (288/692) had received a second dose. Of participants who were offered a vaccine but were not vaccinated, 48% (26/54) reported they decided not to get vaccinated (Appendix Figure 2). Among all participants who had not been offered the mpox vaccine, 75% (421/559) were willing to be vaccinated (Table 3; Appendix Figure 2).

**Table 3:** Mpox vaccine offer and uptake among participants in a survey of mpox diagnosis, behavioral risk modification, and vaccination uptake among gay, bisexual, and other men who have sex with men, United Kingdom, 2022*

Eligibility group	Mpox vaccine offered and vaccinated, no. (%)					Mpox vaccination uptake, % (95% CI)	
	Total offered vaccine	Offered, vaccinated	Offered, not vaccinated	Not offered, not vaccinated		Among those offered vaccine†	All participants‡
Eligible, n = 875	655 (75)	601 (69)	54 (6)§	220 (25)¶		92 (89–94)	69 (65–72)
Not eligible, n = 458	116 (25)	91 (20)	25 (5)	342 (75)		78 (70–86)	20 (16–24)
All participants, n = 1,333	771 (58)	692 (52)	79 (6)#	562 (42)**		90 (88–92)	52 (49–55)

*Participants were eligible for mpox vaccination if they self-reported any of the following: meeting recent male physical sex partners at sex on premises venues, sex parties, or public sex environment (i.e., cruising grounds) since August 2022; >10 recent male physical sex partners since August 2022; recreational drug use associated with chemsex in the past year; recent positive STI test since August 2022; or current HIV PrEP use since December 2021. Percentages may not total 100 due to rounding. PrEP, pre-exposure prophylaxis; SHS, sexual health service, STI, sexually transmitted infection. †Percentage (%) reporting uptake of >1 mpox vaccine dose among those offered an mpox vaccine [(a/(a+b)) × 100%]. ‡Percentage (%) reporting uptake of >1 mpox vaccine dose among all groups [(a/(a+b+c)) × 100%]. §Among participants who were eligible for mpox vaccine eligible and offered a vaccine, but not vaccinated (n = 54), 28 (52%) were waiting to get vaccinated, 26 (48%) decided not to get vaccinated. ¶Among those eligible and not offered, 38% (83/220) had visited an SHS since August 2022 and 57% (126/220) had visited an SHS in the past year. #Among all participants offered an mpox vaccine, but not vaccinated (n = 79), 42 (53%) were waiting to get vaccinated, 37 (47%) decided not to get vaccinated. **Among participants not offered an mpox vaccine, 20% (111/562) had visited an SHS since August 2022 and 32% (182/562) had visited an SHS in the past year.

### Mpox Vaccination Uptake among Vaccine-Eligible Participants

Among GBMSM considered vaccine eligible (66%; n = 875), 75% (n = 655) were offered a vaccine and 601 received vaccination (Table 3; Appendix Table 2, Figure 2). Vaccination uptake was 69% (95% CI 65%–72%) for all eligible participants and 92% (95% CI 89%–94%) for those who were offered a vaccine. Second doses were reported by 41% (252/601) of eligible GBMSM vaccinated; 25% (220/875) of eligible participants were not offered an mpox vaccine, but 77% (168/218) indicated vaccine willingness (Appendix Figure 2). In sensitivity analyses using a lower threshold for multiple partners (>5) to assume vaccine eligibility, we noted a similar level of vaccine uptake (Appendix Table 5).

### Factors Associated with Vaccination

We found evidence of bivariate association with mpox vaccination by age group, sexual orientation, educational qualifications, employment, and financial situation. In adjusted models, bisexual men were less likely to report mpox vaccination (32% vs. 54% of gay or homosexual men; adjusted odds ratio [aOR] 0.43, 95% CI 0.29–0.62), as were participants with below degree-level education qualifications (40% vs. 59% in degree-level or higher; aOR 0.50, 95% CI 0.39–0.63), and unemployed participants (37% vs. 55% of employed participants; aOR 0.59, 95% CI 0.43–0.80) (Appendix Table 2). Participants reporting relationship status as single were more likely to be vaccinated (54% vs. 50% of those in a relationship; aOR 1.27, 95% CI 1.01–1.60). We found no evidence of independent association to age, but we noted the lowest levels of vaccination among persons 16–24 years of age (30% vs. 58% among persons 45–54 years of age; aOR 0.47, 95% CI 0.25–0.87). Persons 16–24 years of age comprised only 5% of mpox vaccinated participants. After adjusting for sociodemographic characteristics, the greatest predictors of mpox vaccination were reporting a positive STI test since August 2022 (aOR 4.09, 95% CI: 2.69–6.22); having an HAV, HBV, or HPV vaccination history (aOR 5.27, 95% CI: 3.72–7.47); reporting a higher (>10 vs. 1) number of physical sex partners since August 2022 (aOR 7.73, 95% CI 5.06–11.8); and reporting PrEP use since December 2021 (aOR 7.09, 95% CI 5.49–9.15). Among mpox vaccinated GBMSM, 87% (601/692) met proxy mpox vaccination eligibility. Participants who met mpox vaccination eligibility were 8 times more likely to have been vaccinated than those who did not meet eligibility (aOR 8.38, 95% CI 6.35–11.1).

In sensitivity analyses examining sociodemographic factors associated with mpox vaccination among vaccine-eligible participants, we found mpox vaccine uptake was less likely among bisexual than gay or homosexual men (aOR 0.49, 95% CI 0.30–0.79), participants with lower educational qualifications (aOR 0.46, 95% CI 0.34–0.63), and unemployed (aOR 0.63, 95% CI 0.42–0.95) (Appendix Tables 2, 4). Those findings were consistent with our analyses of those groups among all participants.

## Discussion

In this large, online survey of GBMSM in the United Kingdom conducted shortly after the 2022 mpox outbreak began, 53% of participants reported adopting a risk modification measure, 75% of eligible participants had been mpox vaccinated, and participants offered a vaccine had very high uptake. Most (87%) participants who were vaccinated met proxy eligibility criteria. Among all 1,333 participants, vaccine uptake was associated with higher levels of sexual risk, suggesting fidelity to targeted vaccination set out by national guidelines during rapid vaccine rollout across the United Kingdom in June 2022. Demographic and behavioral characteristics among participants with mpox-positive tests broadly reflected those described in enhanced surveillance of confirmed cases undertaken by UKHSA (*11*). Among participants who tested mpox-positive, 37% were living with HIV, consistent with high mpox case reporting (*21*–*23*), and 58% vaccine uptake among that group exceeded participant estimates but was subject to small sample size.

We found UK mpox vaccine uptake was similar to levels reported in British Columbia among all (51%) and eligible (66%) transgender persons and GBMSM at sexual health clinics shortly after vaccination implementation (*24*). Although changes to sexual behavior were reported among GBMSM in the United Kingdom during COVID-19 restrictions (*19*,*20*,*25*), limited evidence on behavioral modification in response to the 2022 mpox outbreak is available. Our findings support other evidence of behavioral modification among GBMSM during the mpox outbreak; UK surveillance data show a concurrent, but temporary, decline in lymphogranuloma venereum and *Shigella* species, infections that primarily circulate in the dense, interconnected sexual networks that are also associated with monkeypox transmission (*11*,*26*).

In our study, participants with lower educational qualifications and those without employment reported lower vaccine uptake, differences that we also found in sensitivity analyses restricted to vaccine-eligible participants. Those findings mirrored COVID-19 vaccine uptake inequalities identified in the previous RiiSH survey; during that survey period (December 2021), COVID-19 vaccination was widely accessible in the United Kingdom (*27*). We found bisexual and straight-identifying participants also were less likely to report mpox vaccination, consistent with findings in a smaller cross-sectional study exploring mpox vaccination uptake (*24*). The effects and epidemiology of mpox in bisexual and heterosexual-identifying MSM are unknown. However, a previous systematic review reported lower SHS engagement despite high sexual risk among heterosexual-identifying men who have sex with men (*28*), suggesting a need for tailored vaccination promotion efforts for those groups during mpox resurgence or endemicity, or for other STI outbreaks.

National guidance for targeting mpox vaccination recommended using markers of historical sexual risk associated with STI and HIV incidence (*29*,*30*). Most mpox vaccinated GBMSM met proxy eligibility criteria; however, 31% of those considered vaccine eligible did not report mpox vaccination, most (220/274) of whom did not receive a vaccine offer. That finding could reflect SHS access barriers because only 38% of vaccine-eligible participants who were not offered a vaccine had visited an SHS since August 2022. However, that group might have less regular engagement with SHS (*31*,*32*). Vaccine provision and service-level constraints resulting from increased mpox infection control measures and service reconfigurations that included online triage could have limited face-to-face vaccine offers and subsequent uptake (*33*,*34*). 

To ensure respondent anonymity, the survey did not collect any data that would indicate the region of participant residence; vaccination offer and uptake might have been lower in regions of England that did not experience large outbreaks or where local services did not provide vaccination. Participant-level barriers to vaccination, such as low perceived mpox risk, might also have limited vaccine uptake among eligible participants, especially when mpox incidence fell sharply across the United Kingdom after the July 2022 peak.

Only 41% of mpox-vaccinated participants reported having received a second dose by the survey period, despite availability beginning in September 2022; that finding is similar to uptake reported nationally through May 2023 (*12*). Further exploration of low vaccine course completion is needed, especially because little data exist on the length of protection conferred by a single mpox vaccine dose or complete vaccination (*35*). Although rapid, first-dose vaccination was recommended for outbreak control after favorable efficacy studies (*17*), since September 2022, UKHSA and the Joint Committee on Vaccination and Immunization have recommended 2 mpox vaccine doses for eligible groups (*15*,*36*,*37*).

The RiiSH-Mpox survey was part of a series of repeat, cross-sectional surveys that use consistent methodology and provide key behavioral insights to supplement routine national surveillance data for STIs and HIV. This study provides a timely examination of mpox in a community sample of GBMSM and contextualizes interpretation of the trends in the UK mpox outbreak. However, because of its cross-sectional design, our study is subject to key limitations. First, we cannot determine the time of vaccination in relation to most reported sexual risk behaviors comprising eligibility. Moreover, given the report of behavioral risk modification resulting from the mpox outbreak, vaccine eligibility could be underestimated, and behavior could have changed after vaccine uptake. Second, although observational studies of GBMSM in the United Kingdom and other high-income countries have reported a high mpox vaccine willingness (*38*–*40*), uptake estimates might be subject to sampling and social desirability bias and primarily representative of GBMSM using social media or Grindr. Third, information about survey impressions and click-through rates were not available, limiting insight into survey reach and engagement. Fourth, given higher educational attainment and employment, RiiSH-Mpox participants might represent a more health-literate sample relative to national probability survey estimates in GBMSM (*41*). Prior RiiSH cross-sectional samples reported near universal uptake of complete COVID-19 vaccination (*27*). Thus, although RiiSH-Mpox participants might not be representative of all GBMSM in the United Kingdom, our study sample likely represents key groups targeted for mpox vaccination and vaccination for other sexually transmissible pathogens, such as hepatitis A. Finally, small subgroup sizes across ethnicity and gender identity indicators limit assessment of inequalities in vaccine uptake.

Although high vaccine uptake in eligible GBMSM and adoption of risk modification measures likely led to the reduction of mpox incidence in the United Kingdom in July 2022, the degree of contribution of each measure is unknown. Before and during the reactive vaccine program, timely vaccine resource information distributed and amplified by community-based organizations for GBMSM and other at-risk groups likely contributed to the sharp drop in mpox incidence by the end of 2022 (*9*,*42*). Vaccination implementation across freely accessible and confidential SHS systems across the United Kingdom might have contributed to high uptake.

In conclusion, the use of key behavioral proxies guided mpox vaccination eligibility for GBMSM and aided vaccination implementation for those at risk for mpox in the United Kingdom. Future targeted vaccination rollout should consider rapid, yet equitable provision strategies and engage underserved populations via local outreach and community groups to maximize outreach and vaccine uptake and minimize mpox-related stigma (*43*). Uptake barriers for groups already described to have unmet sexual health needs, such as sexual minority groups and persons with lower social and financial capital (*19*), must be understood to minimize exacerbation of vaccine uptake inequalities. Moreover, because SHS reconfigurations continue, often led by online service expansion, effective vaccination offer and provision strategies for persons using online SHS warrant exploration. Optimizing SHS and outreach-led mpox vaccine offer, as part of a package of preventive interventions for persons with unmet needs, should be considered to maximize the benefit of each sexual health contact. To reduce the likelihood of future mpox outbreaks, given threats of resurgence, provision of first mpox vaccine doses and completion of the vaccination course among those receiving a first dose must be urgently prioritized.

**Appendix.** Additional information on a survey of mpox diagnosis, behavioral risk modification, and vaccination uptake among gay, bisexual, and other men who have sex with men, United Kingdom, 2022.
