# Supplementary material for: Mpox Diagnosis, Behavioral Risk Modification, and Vaccination Uptake among Gay, Bisexual, and Other Men Who Have Sex with Men, United Kingdom, 2022
Source: Emerg Infect Dis. 2024 May;30(5):916–25. doi: 10.3201/eid3005.230676 (PMC11060451; doi:10.3201/eid3005.230676)
Supplement: Supplementary file 1 — Appendix. Additional information on a survey of mpox diagnosis, behavioral risk modification, and vaccination uptake among gay, bisexual, and other men who have sex with men, United Kingdom, 2022. [file 23-0676-Techapp-s1.pdf]

Article DOI: https://doi.org/10.3201/eid3005.230676

EID cannot ensure accessibility for supplementary materials supplied by authors. Readers who have difficulty accessing supplementary content should contact the authors for assistance.

Mpox Diagnosis, Behavioral Risk Modification, and Vaccination Uptake among Gay, Bisexual, and Other Men Who Have Sex with Men, United Kingdom, 2022

Appendix

Appendix Table 1. Sociodemographic and clinical characteristics of RiiSH-Mpox participants with and without an mpox diagnosis history, November–December 2022, 2022*

Characteristics	RiiSH-Mpox participants, no. (%)	Mpox diagnosis history, no. (%)	No mpox diagnosis history, no. (%)	p value
Total	1,333 (100)	35 (100)	1,298 (100)	
Recruitment site				
Facebook	769 (58)	28 (80)	741 (57)	
Grindr	316 (24)	5 (14)	311 (24)	
Twitter	198 (15)	2 (6)	196 (15)	
Instagram	18 (1)	0	18 (1)	
Other	32 (2)	0	32 (2)	0.099
Sociodemographic characteristics				
Median age, y (range; IQR)	45 (16–78; 35–55)	42 (25–58; 38–47)	45 (16–78; 35–55)	
Mean age (SD)	44.8 (12.5)	42.2 (6.9)	44.8 (12.7)	
Age group, y				
16–24	63 (5)	0	63 (5)	
25–34	256 (19)	3 (9)	253 (19)	
35–44	343 (26)	18 (51)	325 (25)	
45–54	327 (25)	12 (34)	315 (24)	
≥55	344 (26)	2 (6)	342 (26)	<0.001
Binary age group, y				
<35	319 (24)	3 (9)	316 (24)	
≥35	1,014 (76)	32 (91)	982 (76)	0.031
Gender identity, sex at birth				
Cisgender male	1,314 (99)	35 (100)	1,279 (99)	
Other gender identity group	19 (1)	0	19 (1)	0.471
Sexual orientation				
Gay or homosexual	1,187 (89)	32 (91)	1,155 (89)	
Bisexual†	146 (11)	3 (9)	143 (11)	0.648
Ethnicity				
White	1,223 (92)	30 (86)	1,193 (92)	
Non-White	110 (8)	5 (14)	105 (8)	0.189
Country of birth				
United Kingdom	1,113 (83)	22 (63)	1,091 (84)	
Outside the United Kingdom	220 (17)	13 (37)	207 (16)	0.001
Nation of residence				
England	1,148 (86)	34 (97)	1,114 (86)	
Scotland, Wales, or Northern Ireland	185 (14)	1 (3)	184 (14)	0.056
Educational level				
Below degree level	495 (37)	7 (20)	488 (38)	
Degree level or higher	838 (63)	28 (80)	810 (62)	0.033
Employment				
Not employed	259 (19)	3 (9)	256 (20)	
Current employment	1,074 (81)	32 (91)	1,042 (80)	0.100

Characteristics	RiiSH-Mpox participants, no. (%)	Mpox diagnosis history, no. (%)	No mpox diagnosis history, no. (%)	p value
Household composition				
Live with others	827 (62)	22 (63)	805 (62)	
Live alone	506 (38)	13 (37)	493 (38)	0.920
Relationship status				
Single	591 (44)	18 (51)	573 (44)	
In a relationship	742 (56)	17 (49)	725 (56)	0.392
Comfortable financial situation‡				
N	693 (52)	12 (34)	681 (52)	
Y	640 (48)	23 (66)	617 (48)	0.034
Clinical and behavioral characteristics				
HIV status				
Negative or unknown	1,131 (85)	22 (63)	1,109 (85)	
Living with HIV	202 (15)	13 (37)	189 (15)	<0.001
HAV vaccination history, >1 dose				
N	430 (32)	8 (23)	422 (33)	
Y	903 (68)	27 (77)	876 (67)	0.228
HBV vaccination history, >1 dose				
N	379 (28)	5 (14)	374 (29)	
Y	954 (72)	30 (86)	924 (71)	0.060
HPV vaccination history, >1 dose				
N	771 (58)	9 (26)	762 (59)	
Y	562 (42)	26 (74)	536 (41)	<0.001
SHS visit in past year				
N	478 (36)	2 (6)	476 (37)	
Y	855 (64)	33 (94)	822 (63)	<0.001
STI tested in past year				
N	405 (30)	1 (3)	404 (31)	
Y	928 (70)	34 (97)	894 (69)	<0.001
STI test positivity since August 2022				
Never STI tested	123 (9)	1 (3)	122 (9)	
Tested before August 2022	513 (38)	7 (20)	506 (39)	
All test results since August 2022 negative or unknown	541 (41)	12 (34)	529 (41)	
>1 Recent positive test result since August 2022	156 (12)	15 (43)	141 (11)	<0.001
Recreational drug use associated with chemsex in past year§				
N	1,222 (92)	27 (77)	1,195 (92)	
Y	111 (8)	8 (23)	103 (8)	0.002
Number of recent male physical sexual partners since August 2022				
0	100 (8)	1 (3)	99 (8)	
1	180 (14)	2 (6)	178 (14)	
2–4	389 (29)	9 (26)	380 (29)	
5–9	280 (21)	6 (17)	274 (21)	
>10	384 (29)	17 (49)	367 (28)	0.094
Met recent male physical sex partners in sex on premises venue, sex party, or PSE since August 2022				
N	888 (67)	17 (49)	871 (67)	
Y	445 (33)	18 (51)	427 (33)	0.022
PrEP use since December 2021¶				
N	534 (47)	2 (9)	532 (48)	
Y	597 (53)	20 (91)	577 (52)	<0.001
Behavior modification to avoid getting mpox since May 2022#				
N	626 (47)	18 (51)	608 (47)	
Y	707 (53)	17 (49)	690 (53)	0.592

*Participants with self-reported mpox test positivity. Those without mpox test positivity (i.e., no diagnosis history) include those without a known positive test (n = 67) and those without a test history (n = 1,231). Percentages may not total 100 due to rounding. HAV, hepatitis A virus; HBV, hepatitis B virus; HPV, human papilloma virus; PSE, public sex environment (i.e., cruising grounds); PrEP, HIV pre-exposure prophylaxis; RiiSH-Mpox, Reducing inequalities in Sexual Health Mpox survey; SHS, sexual health service; STI, sexually transmitted infection.
†Includes those identifying as bisexual, straight, or another orientation.
‡Comfortable includes top 2 quartiles, “I am comfortable” or ”I am very comfortable,” from the question, “How would you best describe your current financial situation.”
§Includes crystal methamphetamine, mephedrone, or gamma-hydroxybutyrate/gamma-butyrolactone.
¶HIV negative or unknown status (n = 1,131); excludes persons living with HIV (n = 202).
#Behavior modification includes self-report of any of the following from May 2022: fewer sexual partners, reduced visits to sex on premises venues or PSE (i.e., cruising grounds), and avoiding: all sex, condomless anal sex, skin-to-skin contact, or visiting clubs or crowds.

Appendix Table 2. Characteristics of, and factors associated with self-reported mpox vaccination uptake among RiiSH-Mpox participants, November–December 2022*

Characteristics	RiiSH-Mpox participants, no. (%)	Mpox vaccinated†				
		No. (%)	OR (95% CI)	p value	aOR (95% CI)‡	p value
Total	1,333 (100)	692 (52)				
Sociodemographic						
Median age, y (range; IQR)	45 (16–78; 35–55)	45 (18–74; 36–54)				
Mean age (SD)	44.8 (12.5)	44.8 (11.7)				
Age-group, y§						
16–24	63 (5)	19 (30)	0.31 (0.17–0.56)		0.47 (0.25–0.87)	
25–34	256 (19)	133 (52)	0.78 (0.56–1.08)		0.79 (0.56–1.11)	
35–44	343 (26)	189 (55)	0.88 (0.65–1.20)		0.86 (0.63–1.18)	
45–54	327 (25)	190 (58)	1.00 Referent		1.00 Referent	
≥55	344 (26)	161 (47)	0.63 (0.47–0.86)	0.0002	0.79 (0.57–1.09)	0.1444
Gender identity, sex at birth						
Cisgender male	1,314 (99)	684 (52)	1.00 Referent		1.00 Referent	
Other orientations	19 (1)	8 (42)	0.67 (0.27–1.68)	0.3885	1.05 (0.40–2.79)	0.922
Sexual orientation						
Gay or homosexual	1,187 (89)	645 (54)	1.00 Referent		1.00 Referent	
Bisexual¶	146 (11)	47 (32)	0.40 (0.28–0.57)	<0.001	0.43 (0.29–0.62)	<0.001
Ethnicity§						
White	1,223 (92)	635 (52)	1.00 Referent		1.00 Referent	
Non-White	110 (8)	57 (52)	1.00 (0.67–1.47)	0.9834	1.06 (0.70–1.61)	0.7668
Country of birth						
United Kingdom	1,113 (83)	566 (51)	1.00 Referent		1.00 Referent	
Outside the United Kingdom	220 (17)	126 (57)	1.30 (0.97–1.73)	0.081	1.17 (0.85–1.59)	0.3374
Nation of residence						
England	1,148 (86)	613 (53)	1.00 Referent		1.00 Referent	
Scotland, Wales, or Northern Ireland	185 (14)	79 (43)	0.65 (0.48–0.89)	0.0069	0.64 (0.46–0.89)	0.0081
Educational qualifications						
Below degree-level	495 (37)	199 (40)	0.47 (0.38–0.59)		0.50 (0.39–0.63)	
Degree-level or higher	838 (63)	493 (59)	1.00 Referent	<0.001	1.00 Referent	<0.001
Employment						
Not employed	259 (19)	97 (37)	0.48 (0.36–0.64)		0.59 (0.43–0.80)	
Current employment	1,074 (81)	595 (55)	1.00 Referent	<0.001	1.00 Referent	0.0007
Household composition						
Lives with others	827 (62)	414 (50)	0.82 (0.66–1.03)		0.81 (0.65–1.03)	
Lives alone	506 (38)	278 (55)	1.00 Referent	0.0834	1.00 Referent	0.084
Relationship status						
Single	591 (44)	320 (54)	1.17 (0.95–1.46)		1.27 (1.01–1.60)	
In a relationship	742 (56)	372 (50)	1.00 Referent	0.1453	1.00 Referent	0.0433
Comfortable financial situation#						
N	693 (52)	331 (48)	0.71 (0.57–0.88)		0.90 (0.71–1.14)	
Y	640 (48)	361 (56)	1.00 Referent	0.0016	1.00 Referent	0.3774
Clinical and behavioral characteristics						
HIV status						
Negative/unknown	1,131 (85)	575 (51)	1.00 Referent		1.00 Referent	
Living with HIV	202 (15)	117 (58)	1.33 (0.98–1.80)	0.0629	1.37 (1.00–1.89)	0.0517
HAV, HBV, or HPV vaccination history**						
N	246 (18)	48 (20)	1.00 Referent		1.00 Referent	
Y	1,087 (82)	644 (59)	6.00 (4.28–8.41)	<0.001	5.27 (3.72–7.47)	<0.001
Behavior modification since May 2022 to avoid getting mpox††						
N	626 (47)	247 (39)	1.00 Referent		1.00 Referent	
Y	707 (53)	445 (63)	1.77 (1.42–2.20)	<0.001	1.63 (1.30–2.04)	<0.001
Mpox vaccination proxy eligibility criteria						
STI test positive since August 2022						
N	1,177 (88)	568 (48)	1.00 Referent		1.00 Referent	
Y	156 (12)	124 (79)	4.15 (2.77–6.23)	<0.001	4.09 (2.69–6.22)	<0.001

Characteristics	RiiSH-Mpox participants, no. (%)	Mpox vaccinated†				
		No. (%)	OR (95% CI)	p value	aOR (95% CI)‡	p value
PrEP use since December 2021 (includes PLWHIV)‡‡						
N	734 (55)	228 (31)	1.00 Referent		1.00 Referent	
Y	599 (45)	464 (77)	7.63 (5.96–9.77)	<0.001	7.09 (5.49–9.15)	<0.001
Recreational drug use associated with chemsex in last year§§						
N	1,222 (92)	617 (50)	1.00 Referent		1.00 Referent	
Y	111 (8)	75 (68)	2.04 (1.35–3.09)	0.0005	2.03 (1.32–3.12)	0.001
No. male physical sex partners since August 2022						
0	100 (8)	31 (31)	1.52 (0.88–2.64)		1.55 (0.88–2.73)	
1	180 (14)	41 (23)	1.00 Referent		1.00 Referent	
2–4	389 (29)	170 (44)	2.63 (1.76–3.93)		2.65 (1.75–4.00)	
5–9	280 (21)	180 (64)	6.10 (3.99–9.34)		6.36 (4.10–9.87)	
≥10	384 (29)	270 (70)	8.03 (5.32–12.1)	<0.001	7.73 (5.06–11.8)	<0.001
≥10 recent male physical sex partners since August 2022						
N	949 (71)	422 (44)	1.00 Referent		1.00 Referent	
Y	384 (29)	270 (70)	2.96 (2.30–3.81)	<0.001	2.78 (2.14–3.62)	<0.001
Met recent male physical sex partners in sex on premises venue, sex party, or PSE since August 2022						
N	888 (67)	382 (43)	1.00 Referent		1.00 Referent	
Y	445 (33)	310 (70)	3.04 (2.39–3.87)	<0.001	2.80 (2.18–3.60)	<0.001
Mpox vaccination eligibility (composite)‡						
N	458 (34)	91 (20)	1.00 Referent		1.00 Referent	
Y	875 (66)	601 (69)	8.85 (6.75–11.6)	<0.001	8.38 (6.35–11.1)	<0.001

*Percentages may not total 100 due to rounding. aOR, adjusted odds ratio; HAV, hepatitis A virus; HBV, hepatitis B virus; HPV, human papilloma virus; OR, odds ratio; PLWHIV, persons living with HIV; PrEP, HIV pre-exposure prophylaxis; PSE, public sex environment (i.e., cruising grounds); SHC, sexual health clinic; STI, sexually transmitted infection.

†Participants with self-reported uptake of ≥1 mpox vaccine dose.

‡Adjusted for age-group, ethnicity, sexual orientation, educational qualifications, employment, and financial situation.

§Selected a priori for model inclusion.

¶Includes those identifying as bisexual, straight, or another orientation.

#Top two quartiles (“I am comfortable”/”I am very comfortable” from the question, “How would you best describe your current financial situation.”

**Participants self-reporting uptake of ≥1 HAV, HBV, or HPV vaccine dose(s).

††Behavior modification includes self-report of any of the following from May 2022: fewer sexual partners, reduced visits to sex on premises venues or PSE (i.e., cruising grounds), and avoiding: all sex, condomless anal sex, skin-to-skin contact, or visiting clubs or crowds.

‡‡Includes people living with HIV (n = 202); those reporting a new HIV diagnosis in the past 5 y were included in questions addressing PrEP use. Among those with a HIV negative/unknown status (n = 1,131), 78% of PrEP users since December 2021 (vs 21% of non-users) reported mpox vaccination (aOR: 12.2 [95% CI: 9.07–16.3]).

§§Includes crystal methamphetamine, mephedrone or gamma-hydroxybutyrate/gamma-butyrolactone. §§Self-report of any mpox vaccination proxy eligibility criteria.

Appendix Table 3. Sociodemographic and clinical characteristics of RiiSH-Mpox participants with and without an mpox diagnosis history that includes self-perceived mpox, November–December 2022*

Characteristics	RiiSH-Mpox participants, no. (%)	Mpox diagnosis history or self-perceived mpox, no. (%)	No mpox diagnosis history or self-perceived mpox, no. (%)	p value
Total	1,333 (100)	52 (100)	1,281 (100)	
Recruitment site				
Facebook	769 (58)	39 (75)	730 (57)	
Grindr	316 (24)	8 (15)	308 (24)	
Twitter	198 (15)	4 (8)	194 (15)	
Instagram	18 (1)	0	18 (1)	
Other	32 (2)	1 (2)	31 (2)	0.134
Sociodemographic characteristics				
Median age, y (range; IQR)	45 (16–78; 35–55)	42 (24–68; 38–47)	45 (16–78; 35–55)	
Mean age (SD)	44.8 (12.5)	42.6 (8.5)	44.8 (12.7)	
Age group, y				
16–24	63 (5)	1 (2)	62 (5)	
25–34	256 (19)	5 (10)	251 (20)	
35–44	343 (26)	25 (48)	318 (25)	
45–54	327 (25)	16 (31)	311 (24)	
≥55	344 (26)	5 (10)	339 (26)	<0.001
Binary age group, y				
<35	319 (24)	6 (12)	313 (24)	
≥35	1,014 (76)	46 (88)	968 (76)	0.033
Gender identity, sex at birth				
Cisgender male	1,314 (99)	52 (100)	1,262 (99)	
Other gender identity group	19 (1)	0	19 (1)	0.376
Sexual orientation				
Gay or homosexual	1,187 (89)	49 (94)	1,138 (89)	
Bisexual†	146 (11)	3 (6)	143 (11)	0.222
Ethnicity				
White	1,223 (92)	45 (87)	1,178 (92)	
Non-White	110 (8)	7 (13)	103 (8)	0.164
Country of birth				
United Kingdom	1,113 (83)	35 (67)	1,078 (84)	
Outside the United Kingdom	220 (17)	17 (33)	203 (16)	0.001
Nation of residence				
England	1,148 (86)	49 (94)	1,099 (86)	
Scotland, Wales, or Northern Ireland	185 (14)	3 (6)	182 (14)	0.084
Educational level				
Below degree level	495 (37)	10 (19)	485 (38)	
Degree level or higher	838 (63)	42 (81)	796 (62)	0.006
Employment				
Not employed	259 (19)	7 (13)	252 (20)	
Current employment	1,074 (81)	45 (87)	1,029 (80)	0.267
Household composition				
Live with others	827 (62)	34 (65)	793 (62)	
Live alone	506 (38)	18 (35)	488 (38)	0.612
Relationship status				
Single	591 (44)	24 (46)	567 (44)	
In a relationship	742 (56)	28 (54)	714 (56)	0.788
Comfortable financial situation‡				
N	693 (52)	21 (40)	672 (52)	
Y	640 (48)	31 (60)	609 (48)	0.088
Clinical and behavioral characteristics				
HIV status				
Negative or unknown	1,131 (85)	36 (69)	1,095 (85)	
Living with HIV	202 (15)	16 (31)	186 (15)	0.001
HAV vaccination history, ≥1 dose				
N	430 (32)	15 (29)	415 (32)	
Y	903 (68)	37 (71)	866 (68)	0.591
HBV vaccination history, ≥1 dose				
N	379 (28)	11 (21)	368 (29)	
Y	954 (72)	41 (79)	913 (71)	0.235
HPV vaccination history, ≥1 dose				
N	771 (58)	17 (33)	754 (59)	
Y	562 (42)	35 (67)	527 (41)	<0.001
SHS visit in past year				
N	478 (36)	6 (12)	472 (37)	

Characteristics	RiiSH-Mpox participants, no. (%)	Mpox diagnosis history or self-perceived mpox, no. (%)	No mpox diagnosis history or self-perceived mpox, no. (%)	p value
Y	855 (64)	46 (88)	809 (63)	<0.001
STI tested in past year				
N	405 (30)	5 (10)	400 (31)	
Y	928 (70)	47 (90)	881 (69)	0.001
STI test positivity since August 2022				
Never STI tested	123 (9)	1 (2)	122 (10)	
Tested before August 2022	513 (38)	14 (27)	499 (39)	
All test results since August 2022 negative or unknown	541 (41)	19 (37)	522 (41)	
>1 Recent positive test result since August 2022	156 (12)	18 (35)	138 (11)	<0.001
Recreational drug use associated with chemsex in past year§				
N	1,222 (92)	41 (79)	1,181 (92)	
Y	111 (8)	11 (21)	100 (8)	0.001
Number of recent male physical sexual partners since August 2022				
0	100 (8)	1 (2)	99 (8)	
1	180 (14)	3 (6)	177 (14)	
2–4	389 (29)	12 (23)	377 (29)	
5–9	280 (21)	11 (21)	269 (21)	
>10	384 (29)	25 (48)	359 (28)	0.015
Met recent male physical sex partners in sex on premises venue, sex party, or PSE since August 2022				
N	888 (67)	24 (46)	864 (67)	
Y	445 (33)	28 (54)	417 (33)	0.001
PrEP use since December 2021¶				
N	534 (47)	6 (17)	528 (48)	
Y	597 (53)	30 (83)	567 (52)	<0.001
Behavior modification to avoid getting mpox since May 2022#				
N	626 (47)	26 (50)	600 (47)	
Y	707 (53)	26 (50)	681 (53)	0.654

*Participants with self-reported mpox test positivity. Those without mpox test positivity (i.e., no diagnosis history) include those without a known positive test (n = 67) and those without a test history (n = 1,231). Percentages may not total 100 due to rounding. HAV, hepatitis A virus; HBV, hepatitis B virus; HPV, human papilloma virus; PSE, public sex environment (i.e., cruising grounds); PrEP, HIV pre-exposure prophylaxis; RiiSH-Mpox, Reducing inequalities in Sexual Health Mpox survey; SHS, sexual health service; STI, sexually transmitted infection.
†Includes those identifying as bisexual, straight, or another orientation.
‡Comfortable includes top 2 quartiles, "I am comfortable" or "I am very comfortable," from the question, "How would you best describe your current financial situation."
§Includes crystal methamphetamine, mephedrone, or gamma-hydroxybutyrate/gamma-butyrolactone.
¶HIV negative or unknown status (n = 1,131); excludes persons living with HIV (n = 202).
#Behavior modification includes self-report of any of the following from May 2022: fewer sexual partners, reduced visits to sex on premises venues or PSE (i.e., cruising grounds), and avoiding: all sex, condomless anal sex, skin-to-skin contact, or visiting clubs or crowds.

Appendix Table 4. Sociodemographic, clinical, and behavioral characteristics of, and factors associated with self-reported mpox vaccination uptake among RiiSH-Mpox participants assumed eligible for mpox vaccination, November–December 2022*

Characteristics	Participants presumed vaccine eligible, no. (%)	Mpox vaccinated†				
		No. (%)	OR (95% CI)	p value	aOR (95% CI)‡	p value
Total	875 (100)	601 (69)				
Sociodemographic characteristics						
Median age, y (range; IQR)	45 (17–78; 36–54)	45 (18–74; 36–53)				
Mean age (SD)	44.9 (12.0)	44.7 (11.7)				
Age group, y§						
16–24	29 (3)	16 (55)	0.44 (0.20–0.97)		0.57 (0.25–1.33)	
25–34	164 (19)	117 (71)	0.89 (0.56–1.39)		0.88 (0.55–1.40)	
35–44	243 (28)	166 (68)	0.77 (0.51–1.15)		0.72 (0.48–1.09)	
45–54	221 (25)	163 (74)	1.00 Referent		1.00 Referent	
>55	218 (25)	139 (64)	0.63 (0.42–0.94)	0.0915	0.76 (0.50–1.18)	0.4711
Gender identity, sex at birth						
Cisgender male	862 (99)	594 (69)	1.00 Referent		1.00 Referent	
Other gender identity group	13 (1)	7 (54)	0.53 (0.18–1.58)	0.2603	0.75 (0.24–2.38)	
Sexual orientation						

		Mpox vaccinated†				
Characteristics	Participants presumed vaccine eligible, no. (%)	No. (%)	OR (95% CI)	p value	aOR (95% CI)‡	p value
Gay or homosexual	795 (91)	559 (70)	1.00 Referent		1.00 Referent	
Bisexual¶	80 (9)	42 (53)	0.47 (0.29–0.74)	0.0015	0.49 (0.30–0.79)	0.0037
Ethnicity§						
White	802 (92)	552 (69)	1.00 Referent		1.00 Referent	
Non-White	73 (8)	49 (67)	0.92 (0.55–1.54)	0.7646	1.01 (0.59–1.73)	0.9749
Country of birth						
United Kingdom	721 (82)	492 (68)	1.00 Referent		1.00 Referent	
Outside the United Kingdom	154 (18)	109 (71)	1.13 (0.77–1.65)	0.081	1.03 (0.68–1.54)	0.8998
Nation of residence						
England	760 (87)	528 (69)	1.00 Referent		1.00 Referent	
Scotland, Wales, or Northern Ireland	115 (13)	73 (63)	0.76 (0.51–1.15)	0.2015	0.75 (0.49–1.14)	0.1821
Educational level						
Below degree level	291 (33)	165 (57)	0.44 (0.33–0.60)		0.46 (0.34–0.63)	
Degree level or higher	584 (67)	436 (75)	1.00 Referent	<0.001	1.00 Referent	<0.001
Employment						
Not employed	144 (16)	80 (56)	0.50 (0.35–0.73)		0.63 (0.42–0.95)	
Current employment	731 (84)	521 (71)	1.00 Referent	0.0003	1.00 Referent	0.0286
Household composition						
Live with others	527 (60)	358 (68)	0.92 (0.68–1.23)		0.91 (0.67–1.24)	
Live alone	348 (40)	243 (70)	1.00 Referent	0.5535	1.00 Referent	0.5565
Relationship status						
Single	397 (45)	281 (71)	1.20 (0.90–1.60)		1.24 (0.92–1.68)	
In a relationship	478 (55)	320 (67)	1.00 Referent	0.2227	1.00 Referent	0.1571
Comfortable financial situation#						
N	420 (48)	278 (66)	0.80 (0.60–1.07)		1.00 (0.74–1.35)	
Y	455 (52)	323 (71)	1.00 Referent	0.1263	1.00 Referent	0.9896
Clinical and behavioral characteristics						
HIV status						
Negative or unknown	759 (87)	523 (69)	1.00 Referent		1.00 Referent	
Living with HIV	116 (13)	78 (67)	0.93 (0.61–1.41)	0.7197	1.02 (0.66–1.58)	0.9269
HAV, HBV, or HPV vaccination history**						
N	91 (10)	32 (35)	1.00 Referent		1.00 Referent	
Y	784 (90)	569 (73)	4.88 (3.09–7.71)	<0.001	4.42 (2.74–7.13)	<0.001
Behavior modification to avoid getting mpox since May 2022††						
N	398 (45)	246 (62)	1.00 Referent		1.00 Referent	
Y	477 (55)	355 (74)	1.80 (1.35–2.40)	0.0001	1.63 (1.21–2.20)	0.0013

*Percentages may not total 100 due to rounding. Mpox vaccine eligibility was defined as the report of any of the following since August 2022: ≥10 physical male sex partners; meeting any physical male sex partners in a sex on premises venue, sex party, or PSE (i.e., cruising grounds); a positive STI test; or in the last year, report of: PrEP use (as a proxy for those at risk of mpox acquisition); or use of recreational drugs associated with chemsex (crystal methamphetamine, mephedrone or gamma-hydroxybutyrate/gamma-butyrolactone). HAV, hepatitis A virus; HBV, hepatitis B virus; HPV, human papilloma virus; RiiSH-Mpox, Reducing inequalities in Sexual Health Mpox survey.

†Participants with self-reported uptake of ≥1 mpox vaccine dose.

‡Adjusted for age-group, ethnicity, sexual orientation, educational qualifications, employment, and financial situation.

§Selected a priori for model inclusion.

¶Includes those identifying as bisexual, straight, or other orientation.

#Comfortable includes top 2 quartiles, "I am comfortable" or "I am very comfortable," from the question, "How would you best describe your current financial situation."

**Participants self-reporting uptake of ≥1 HAV, HBV, or HPV vaccine dose.

††Behavior modification includes self-report of any of the following from May 2022: fewer sexual partners, reduced visits to sex on premises venues or public sex environment (i.e., cruising grounds), and avoiding: all sex, condomless anal sex, skin-to-skin contact, or visiting clubs or crowds.

Appendix Table 5. Mpox vaccine offer and uptake among RiiSH-Mpox participants considered eligible for mpox vaccination (sensitivity analysis, ≥5 recent male physical sex partners), November–December 2022*

Participants	Total offered vaccine, no. (%)	Mpox vaccine offered and vaccinated, no. (%)			Mpox vaccination uptake, % (95% CI)	
		Offered, vaccinated	Offered, not vaccinated	Not offered, not vaccinated	Among those offered vaccine†	All participants‡
Vaccine-eligible, n = 951	685 (72)	626 (66)	59 (6)	266 (28)	91 (89–93)	66 (63–69)
All participants, n = 1,333	771 (58)	692 (52)	79 (6)	562 (42)	90 (88–92)	52 (49–55)

*Participants were eligible for mpox vaccination if they self-reported any of the following: meeting recent male physical sex partners at sex on premises venues, sex parties, or public sex environment (i.e., cruising grounds) since August 2022; >10 recent male physical sex partners since August 2022; recreational drug use associated with chemsex in the past year; recent positive STI test since August 2022; or current HIV PrEP use since December 2021. Percentages may not total 100 due to rounding. PrEP, pre-exposure prophylaxis; RiiSH-Mpox, Reducing inequalities in Sexual Health Mpox survey; SHS, sexual health service, STI, sexually transmitted infection.
†Percentage (%) reporting uptake of ≥1 mpox vaccine dose(s) among those offered an mpox vaccine [(a/(a+b))*100%].
‡Percentage (%) reporting uptake of ≥1 mpox vaccine dose(s) among all group [(a/(a+b+c)) *100%].

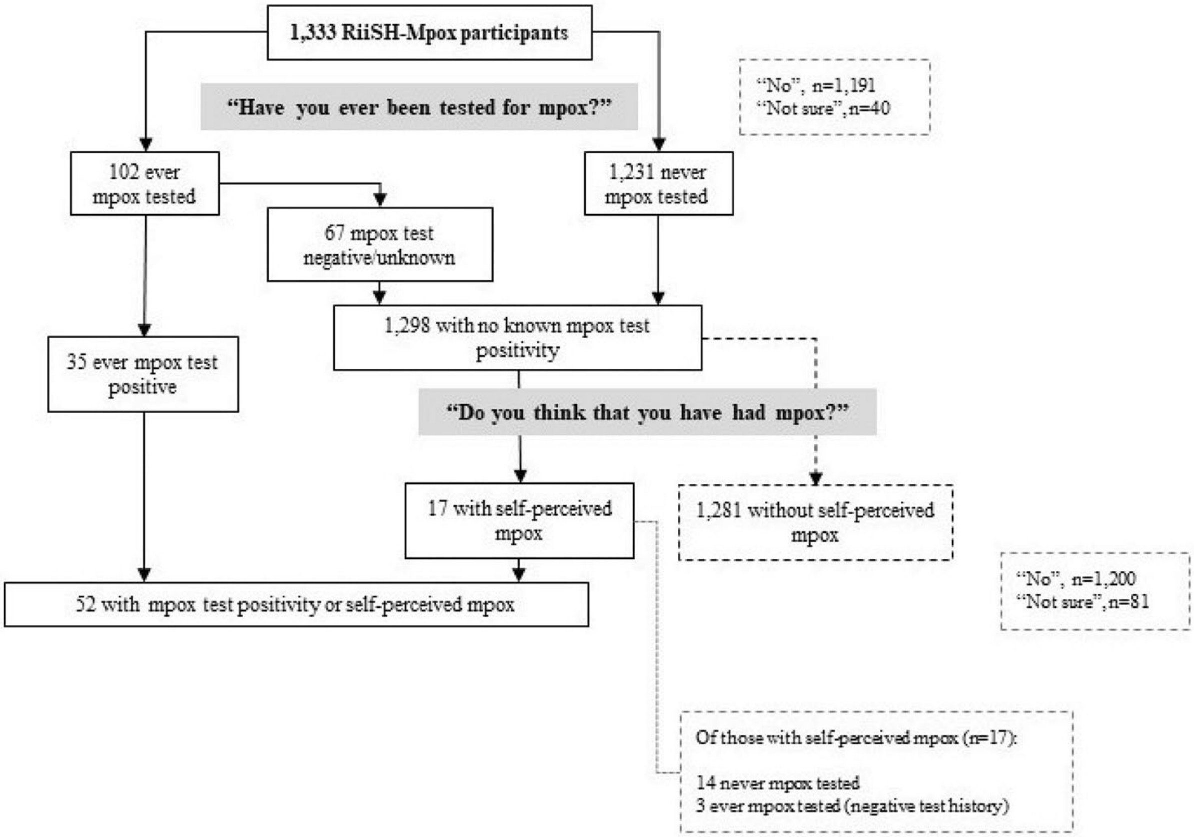

Appendix Figure 1. Self-reported mpox positivity and self-perceived mpox in RiiSH-Mpox participants, November–December 2022. RiiSH-Mpox, Reducing inequalities in Sexual Health Mpox survey.

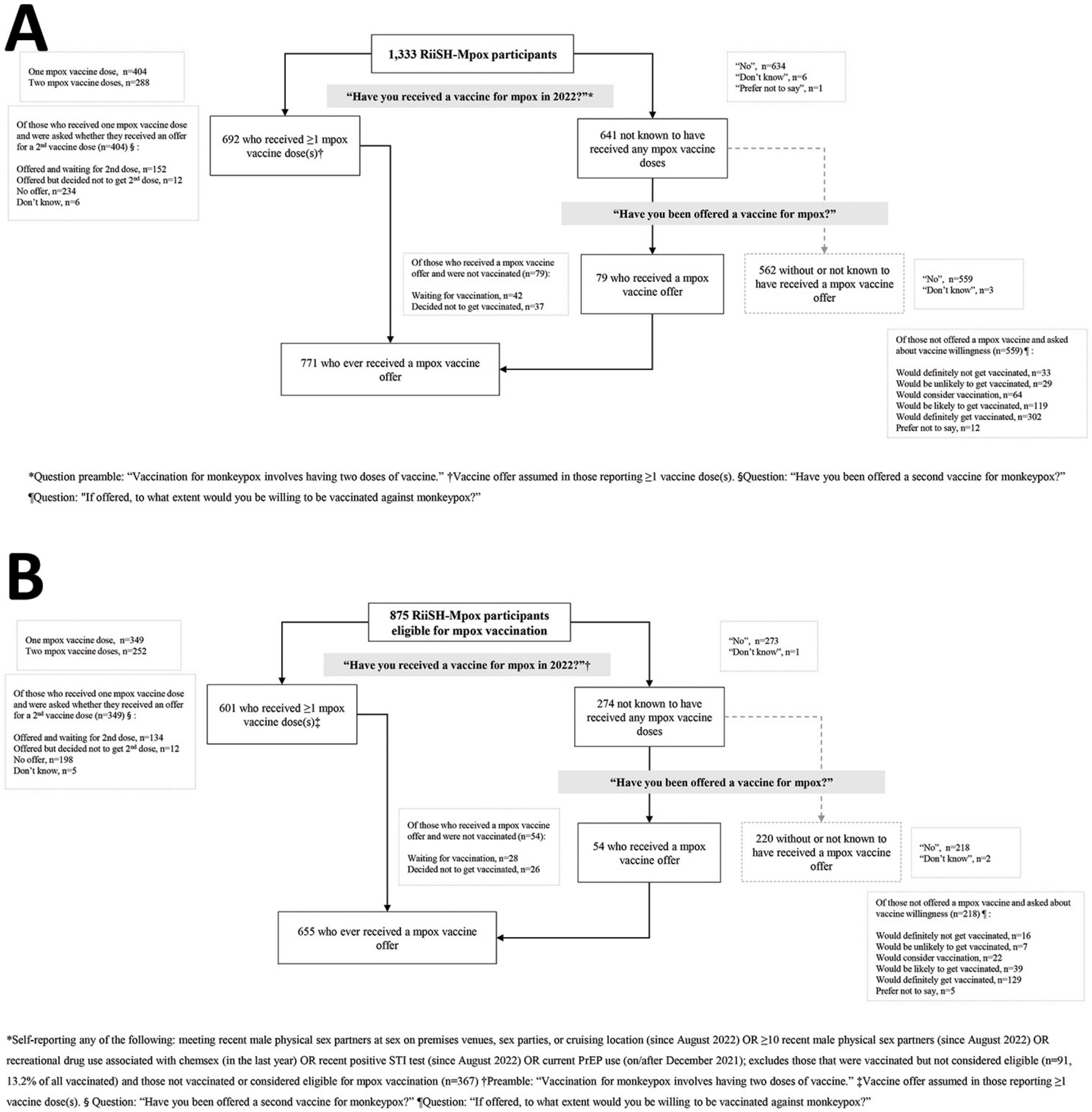

Appendix Figure 2. Mpox vaccination and offer reported in all RiiSH-Mpox participants and those considered mpox vaccine eligible, November–December 2022. RiiSH-Mpox, Reducing inequalities in Sexual Health Mpox survey. A) All RiiSH-Mpox participants. *Question preamble: "Vaccination for monkeypox involves having two doses of vaccine." †Vaccine offer assumed in those reporting ≥1 vaccine dose(s). §Question: "Have you been offered a second vaccine for monkeypox?" ¶Question: "If offered, to what extent would you be willing to be vaccinated against monkeypox?" B) All RiiSH-Mpox vaccine eligible participants. *Self-reporting any of the following: meeting recent male physical sex partners at sex on premises venues, sex parties, or PSE (i.e. cruising grounds) (since August 2022) OR ≥10 recent male physical sex partners (since August 2022) OR recreational drug use associated with chemsex (in the last year) OR recent positive STI test (since August 2022) OR current PrEP use (on/after December 2021); excludes those that were vaccinated but not considered eligible (n=91, 13.2% of all vaccinated) and those

not vaccinated or considered eligible for mpox vaccination (n=367) †Preamble: “Vaccination for monkeypox involves having two doses of vaccine.” ‡Vaccine offer assumed in those reporting ≥1 vaccine dose(s). §Question: “Have you been offered a second vaccine for monkeypox?” ¶Question: “If offered, to what extent would you be willing to be vaccinated against monkeypox?”
